# A national survey of ethnic differences in knowledge and understanding of supplementary health insurance

**DOI:** 10.1186/s13584-017-0137-4

**Published:** 2017-03-07

**Authors:** Manfred S. Green, Samah Hayek, Jalal Tarabeia, Mohammad Yehia, Neta HaGani

**Affiliations:** 1 School of Public Health, University of Haifa, Abba Hushi 199, Haifa, Israel; 2 Faculty of Nursing, The Yezreel Valley College, Afula, Israel; 3 Triangle Research and Development Center, Kfar Kara, Israel

## Abstract

**Background:**

Knowledge and understanding of what health insurance covers is an important public health issue. In Israel, whereas national health insurance covers all residents, optional supplemental health insurance (SHI) can be purchased from the healthcare providers, for additional, special services. The purpose of this study was to identify disparities between Jews and Arabs in their knowledge and understanding of SHI.

**Methods:**

National, cross-sectional, telephone survey using a structured questionnaire, among random samples of 814 Jews and 800 Arabs. Knowledge and understanding of health insurance was assessed by a score based on correct answers to 8 questions. Log-linear regression was used to estimate association between health insurance knowledge and population group, after controlling for potential confounding independent variables.

**Results:**

Ninety one percent of Jews and 62% of Arabs reported owning SHI. Among both groups, knowledge levels were low on a 0–8 scale. However, the average score for Jews was statistically higher (Mean = 3.50, S.D = 1.69) as compared with Arabs (Mean = 2.78, S.D = 1.70) (*p* < 0.001). The adjusted health insurance knowledge score was significantly higher among Jews than Arabs (Prevalence ratio = 1.10; 95% CI = 1.06–1.13), indicating that differences remain even after controlling for socio-demographic characteristics and SHI ownership.

**Conclusions:**

There is a large gap between the public’s understanding of what is covered by SHI and the services that it covers in practice. Low SHI knowledge and understanding may lead to frustration, and limit access to additional health care among populations that suffer from socio-economic inequalities. These findings emphasize the need to provide clearer and more culturally sensitive information on health insurance coverage.

**Electronic supplementary material:**

The online version of this article (doi:10.1186/s13584-017-0137-4) contains supplementary material, which is available to authorized users.

## Background

Knowledge and understanding of health insurance coverage is important to exercise rights to good health care. When deciding whether to purchase health insurance, consumers struggle with calculating their costs and with medical terminology [1]. Consumers also often have difficulties in weighing complex information [2] and show limited ability to weigh the co-payments against insurance premium costs [3]. Although many experience difficulties understanding and thus operating in the health insurance market, relatively little attention has been paid in the literature to knowledge and understanding of health insurance coverage [3].

National health insurance (NHI), introduced in Israel in 1995, covers a comprehensive package of medical services including primary and specialist medical consultations, medications, hospitalization and surgical procedures. It is funded through compulsory taxation and is calculated according to the level of income and social security status (salaried/self/un-employed or student). Medical services are obtained through membership in one of four health funds, which are similar in many ways to health maintenance organizations (HMO).

In addition to the basic package covered by NHI, supplementary health insurance (SHI) is offered to all members of the health funds at additional cost. It covers items such as discounts on special services not included in the basic package, more options for second opinions, alternative medicine, cosmetic procedures, discounts on dental care etc. There are also special plans for women, children, young/old people and families, such as treatment for childhood developmental problems and discounts on orthodontic care [4]. SHI can be purchased at several levels of comprehensiveness and costs. All members are eligible for SHI and there is no selection based on age or health status, although the costs of SHI plans increase with age. In addition to SHI provided by health funds, consumers can purchase commercial health insurance (CHI) either directly from private non-health fund related insurance companies or as groups through their workplace or through membership in professional organizations.

In spite of universal healthcare, there are inequalities between Arabs and Jews in health and health care which may be related to gaps in knowledge and utilization of supplementary health insurance. The Arab population has poorer health status compared to the Jewish population. Arabs have higher rates of heart diseases and diabetes and lower life expectancy [5, 6]. In addition, the Arab population is characterized by lower education and income and, larger families and tend to reside in peripheral areas where the access to health professionals and services are lower [7]. Together with language and cultural barriers, all of these factors may contribute to inequalities in health [8].

In 2009, 81% of the adult population in Israel was insured by some kind of additional health insurance (i.e. either SHI or CHI). This rate was lower among Arab-speakers (63%) and low-income groups (66%). Thirty five percent reported they had CHI [9].

In efforts to promote accessibility to information about services and rights, the Ministry of Health (MOH) operates a website with information regarding SHI. In addition, in 2011, the MOH established a telephone service center which provides information in 5 languages on matters such as: rights in the health system and the National Health Insurance Law [10].

While efforts are being made to provide the public with information on SHI coverage, it is not clear to what extent various segments of the population understand what additional services are included and whether they actually need them. If they fail to understand the kind of services provided by SHI, this could lead to a number of consequences. Firstly, there may be excessive outlays for insurance for services they do not need. Secondly, there may be disappointment resulting from the gap between the expected and actual services covered by SHI. Thirdly, there may be under-insurance for services needed. Finally, there may be underutilization of the services provided by SHI when needed, due to lack of understanding of the services covered by the insurance [11]. In Israel, there is a paucity of data on the extent to which the population understands the coverage provided by SHI. Moreover, health insurance knowledge may differ between the Arab and Jewish population groups due to cultural and language differences.

The overall objective of this study was to compare the knowledge and understanding of a number of components of SHI between Israeli Arabs and Jews. The subjects studied were coverage for private nursing, choice of physician, second opinion, private room in the hospital, reduced waiting times, lower out-of-pocket expenses, catastrophic illnesses, treatment abroad, transplants, special diagnostic tests and in general, higher availability of services.

## Methods

The study was carried out in two phases. In the first phase, six focus groups were conducted, each comprising six to eight men and women, three in each of the Jewish and Arab populations. The members of the focus groups were recruited from the general population and participants were asked in-depth questions about their understanding and expectations from SHI. The questions and responses were recorded and the transcript summarized into major categories and themes. These were used to finalize the contents of the questionnaire.

The second phase of this study comprised two population-based cross-sectional telephone surveys. The sampling frame comprised of the adult (age 25–75) Arab and Jewish population in Israel. The sampling was random and those who were unable to complete a telephone interview were excluded from the study. The surveys were conducted by a survey company, using trained interviewers and random digit dialing. Unanswered numbers were removed from the sample frame after 8 attempts. Each interview lasted about 10 min. Quality control procedures were implemented at all levels of data collection (training of staff, re-interviewing a sub-sample), data entry and data analysis. In total, 1614 full interviews and 158 partial interviews were conducted, 149 of those contacted found it difficult to respond and 868 refused to participate.

The justification of the sample size is as follows: For the prevalence estimates, such as ownership of SHI, and expectations from the services provided by SHI, the sample sizes were selected to obtain precision based on 95% confidence intervals of ±4%, allowing for an expected percentage of 60% for SHI ownership, yields sample sizes of about 550 in each group. For the comparison of prevalence between sub-groups, the sample sizes were chosen to detect differences between the groups of at least 5% in the prevalence of estimates for variables such as ownership of SHI and expectations from SHI, with alpha chosen to be 5% and a statistical power of 80%. This required sample sizes of about 500 in each group. For multivariate analyses the sample sizes needed to be increased to account for the additional variables in the analyses. Thus the sample sizes were increased to 800 in each group.

The questionnaires covered four areas: 1) SHI knowledge 2) behaviors and utilization of SHI 3) attitudes and expectations from SHI services 4) reasons for purchasing SHI. The questionnaires were translated into Hebrew, Arabic and Russian and back-translated, and tested in the respective communities on samples of about 20 people from the target populations. The questionnaires were tested for face, content and consensual validity, for retest reliability (correlations) and internal consistency (Cronbach’s alpha). The final questionnaire is attached in Additional file 1.

For the questionnaire survey, the main dependent variable was knowledge of health insurance based on a score from the sum of the correct answers to 8 questions (1 = true, 0 = not true/don’t know) on services that may or may not be covered by SHI such as: “private room in a hospital”, consultation with a specialist, medications not included in the standard basket of services (items 16–23), generating a summary score ranging from 0 to 8 (α = 0.75). Other dependent variables were based on an average score on a 1–5 Likert scale (1 = do not agree, 5 = very much agree) and included: considerations for purchasing SHI (items 10–13) (α = 0.57), ways of obtaining knowledge of health related issues such as: health insurance, disease prevention and treatment (items 39–43) (α = 0.60) and frequency of obtaining knowledge about SHI (item 44). Participants were also asked about reasons for not purchasing SHI (item 3).

The main independent variable was population group (Jews, Arabs). Potential confounding variables included age, sex, education (primary, secondary, technical and academic), socioeconomic status-SES (based on family income), health fund membership (one of four possible health funds), health status (self-reported on a Likert scale 1–5) and occupation (salaried or self-employed).

Standard univariate analyses were conducted to describe the characteristics of the study population by socio-demographic and other relevant descriptive variables. The normal approximation was used to provide relevant confidence intervals (CIs). In order to assess the factors associated with the SHI knowledge score, we used log-linear regression with negative binomial distribution to adjust for the over dispersed answers. Prevalence ratios (PR) were computed with 95% CIs. All the statistical analyses were conducted using SAS (version 9.3. Cary, NC: SAS Institute Inc; 2011).

## Results

The characteristics of the study sample are given in Table 1. There were more women than men in both groups. Compared to the Jewish respondents, Arab respondents were younger (mean age 47.0 vs. 50.6), with less education, more children per family, lower household income, more likely to be married and had poorer self-reported health status. There were similarities between the two groups in prevalence of chronic disease and chronic use of medications (Table 1). In general, characteristics of each sample by gender, age, and education, were similar to their proportion among each population group in Israel [12].

**Table 1:** Ethnic difference in demographic characteristics of the study population

		Arabs	Jews	*P* value
		(*N* = 800)	(*N* = 814)	
		n, %	n, %	
Age (years)	Mean (STD)	47.0 (11.9)	50.6 (12.5)	*P* < 0.001
	Min-Max	[25–75]	[25–75]	
Gender				*P* = 0.853
	Males	358 (44.8%)	368 (45.2%)	
	Females	442 (55.2%)	446 (54.8%)	
Marital status				*P* < 0.001
	Single/Divorced/Separated/Single parent/Widow	103 (12.9%)	137 (16.9%)	
	Married/live with a partner	696 (87.1%)	674 (83.1%)	
Number of children less than 18 years	Mean (STD)	1.56 (1.6)	1.44 (1.8)	*P* = 0.169
	[Min-Max]	[0–10]	[0–10]	
Employment				*P* < 0.001
	Salaried	362 (45.4%)	512 (63.0%)	
	Self employed	90 (11.3%)	89 (11.0%)	
	Pensioner	64 (8.0%)	106 (13.0%)	
	Other^a^	282 (35.3%)	105 (12.9%)	
Household income				
	Below average	405 (53.4%)	211 (30.9%)	
	Average	196 (25.8%)	166 (24.3%)	
	Above average	158 (20.8%)	305 (44.7%)	
Education				*P* < 0.001
	Less than high school	325 (50.0%)	103 (12.8%)	
	High school –matriculation	151 (19.0%)	116 (14.4%)	
	Academic	269 (33.9%)	454 (56.3%)	
	Professional and others	48 (6.0%)	134 (16.6%)	
Chronic disease				*P* = 0.801
	Yes	219 (27.4%)	224 (28.0%)	
	No	580 (72.6%)	577 (72.0%)	
Medication				*P* < 0.001
	Yes	307 (38.4%)	328 (40.7%)	
	No	492 (61.6%)	477 (59.2%)	
Overall health status				*P* < 0.001
	Bad	57 (7.2%)	37 (4.6%)	
	Good	159 (20.0%)	112 (14.0%)	
	Very Good	577 (72.8%)	651 (81.4)	
Owning SHI				
	Yes	497(62.1%)	743(91.3%)	*P* < 0.001
	No	303(37.9%)	71(8.7%)	

^a^“Other” Include: Stay at home parents, students and unemployed

### SHI ownership, costs and utilization

**Fig. 1:**
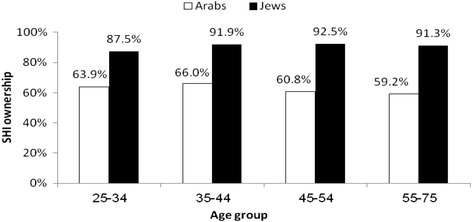
Percent of SHI owners by ethnic group and age

Ninety one percent of the Jews in the sample reported owning SHI as compared with 62% of the Arabs. In addition, 35% of the Jews and 52.5% of the Arabs declared that they had either never used or had no recollection of using their SHI. In Fig. 1 the distribution of SHI ownership is shown by age and ethnic group. About 28% of Jews and 39% of Arabs considered the outlay for SHI to be a significant part of household expenses. The average cost for SHI estimated by Jews was 247 NIS per month whereas Arabs estimated it at 200 NIS per month. About 40% of Jews and 56% of Arabs believed that SHI cost less than 200 NIS per month. Among the main reasons for not purchasing SHI were: high costs (28%) and having NHI providing all necessary services (27%). Only 7% reported having CHI as a reason for not purchasing SHI (Data is not shown in a table).

### Reasons for purchasing SHI

There were no significant differences between Jews and Arabs in the average score (1–5 Likert scale, 1 = do not agree; 5 = very much agree) of purchasing SHI due to better high standard treatment (3.43 vs. 3.50, respectively), fear from catastrophic illnesses not covered by the NHI (3.65 vs. 3.75, respectively), and subsidized medications and treatments (3.81 vs. 3.95). However, there were significant differences between Jews and Arabs in the average score of purchasing SHI due to shorter waiting list (2.66 vs. 3.02, respectively) (Data is not shown in a table).

### Knowledge about SHI

Knowledge about SHI is shown in Table 2. Participants were presented with different health care services and were asked to identify which services they think are included in SHI coverage. Items were scored dichotomously as correct, incorrect or “don’t know”.

**Table 2:** Familiarity with SHI according to knowledge about Services that are included or not included in SHI

	Arabs (800)		Jews (814)		*P* value^1^
	n, %		n, %		
Subsidized Private Nurse in hospitalization after surgery					*P* = 0.996
Included^a^	189	23.7	192	23.7	
Not Included	226	28.3	259	31.9	
Don’t know	383	48.0	360	44.4	
Choosing a doctor or a surgeon from every hospital in Israel					*P* = 0.780
Included	375	46.9	465	57.3	
Not included^a^	175	21.9	173	21.3	
Don’t know	249	31.2	173	21.3	
Subsidized private prenatal care and fertility treatments					*P* = 0.628
Included^a^	386	48.3	373	47.0	
Not included	122	15.3	82	10.3	
Don’t know	292	36.5	338	42.6	
Subsidized transplants and surgeries abroad					*P* = 0.010
Included^a^	357	44.7	413	51.1	
Not included	165	20.7	191	23.6	
Don’t know	277	34.7	205	25.3	
Private room in different hospitals in Israel					*P* < 0.001
Included	146	18.3	78	9.7	
Not included^a^	329	41.2	466	57.8	
Don’t know	324	40.6	262	32.5	
Cancer Medications not covered by basic health insurance					*P* = 0.196
Included	269	33.7	257	31.8	
Not included^a^	190	23.8	215	26.6	
Don’t know	339	42.5	336	41.6	
Partial coverage of Consultation with a specialist					*P* < 0.001
Included^a^	350	43.9	670	82.8	
Not included	188	23.6	50	6.2	
Don’t know	259	32.5	89	11.0	
Full coverage of Private physiotherapy					*P* < 0.001
Included	247	31.2	178	22.1	
Not included^a^	250	31.6	346	43.0	
Don’t know	295	37.2	281	34.9	

^a^Correct answer

^1^ *p*-value was calculated for incorrect answer together with “don’t know” in comparison with correct answers

Regarding some of the services, a large percentage of participants reported they “don’t know” if they are included in SHI. For example, many of the Arabs (48%) and the Jews (44%) didn’t know that SHI subsidize services such as a private nurse during hospitalization and do not cover fully other services such as physiotherapy (37% Arabs and 35% Jews). Many Arabs (43%) and Jews (42%) did not know that SHI does not cover cancer medications not included in the basket of services covered by NHI because SHI does not cover treatment for life threatening illnesses.

In addition, many participants wrongly indicated certain services as included in SHI when they were not. For example: most of the participants presumed that SHI gives them the option to select a surgeon or physician of one’s choice from anywhere in the country (46% Arabs and 57% Jews), although SHI only offers a limited choice of doctors/surgeons from a list provided by the health fund [13].

The differences between Jews and Arabs were both in knowledge about services that are partially covered such as consultation with a specialist (44% for Arabs and 83% for Jews) and about services that are not covered such as full coverage for physiotherapy (31% for Arabs and 22% for Jews) and having a private room during hospitalization (18% for Arabs and 10% for Jews). For prenatal care, the knowledge was similar between the two groups, whereas for subsidized surgery abroad, there was slightly less knowledge among Arabs (see Table 2). Overall, the scores for knowledge were lower for Arabs than for Jews (2.78 vs. 3.49, *p* < 0.001) (Data is not shown in a table).

The results of the multivariate regression analyses are shown in Table 3. Jews had a significantly high score for health insurance knowledge (PR = 1.12; CI 1.06–1.13) after controlling for potential confounders. In order to assess whether ownership of SHI affected the difference in the health insurance knowledge score between Jews and Arabs, we carried out further regression analyses (Table 3). After controlling for ownership of SHI, The PR changed from 1.12 to 1.10 which was still statistically significant (PR = 1.10; CI 1.06–1.13, *p *< 0.001).

**Table 3:** Estimates of the prevalence ratios for the association between Ethnicity and Supplementary Health Insurance knowledge score – Results of the Log linear regression (Negative-Binomial Regression)

		PR (95% CI)	*P*
Ethnicity			
	Jews vs. Arabs	1.10 (1.06–1.13)	*P* < 0.001
Age		0.99 (0.94–0.99)	*P* < 0.001
Gender	Females vs. Males	0.96 (0.94–0.99)	*P* = 0.003
Education			*P* < 0.001
	Less equal high school vs. professional	0.93 (0.88–0.98)	*P* = 0.012
	High school- matriculation vs. professional	1.04 (0.98–1.11)	*P* = 0.161
	Academic degree vs. professional	1.12(1.07–1.20)	*P* < 0.001
Job			*P* = 0.410
	Employed vs. not in labor force	1.01 (0.96–1.06)	*P* = 0.838
	Self employed	0.99 (0.92–1.07)	*P* = 0.453
	Pensioner	1.05 (0.97–1.14)	*P* = 0.153
Marital status			*P* = 0.439
	Married vs. single	1.03 (0.98–1.07)	
Number of children less than 18 yrs. Old		1.02 (0.99–1.03)	*P* = 0.051
Chronic disease	Yes vs. No	1.03 (0.99–1.07)	*P* = 0.120
Medication	Yes vs. No	0.99 (0.95–1.03)	*P* = 0.670
Owning SHI	Yes vs. No	1.10(1.06–1.15)	*P* < 0.001

### Attempts to learn about SHI

More than 50% of both Arabs and Jews reported that they lacked information on SHI.

A large percentage did not investigate what SHI covers before purchasing it (36% among Jews and 32% among Arabs). About a third of participant (32%) reported they don’t know at all what their monthly cost of SHI is and relatively few reported that they knew exactly what it cost (19%).

Among Jews, friends/family and the internet/social media were rated as most important for obtaining knowledge regarding health related issues (such as: health insurance, disease prevention and treatment). Among Arabs, healthcare professionals (doctors/nurses) and television/radio were the most important for obtaining knowledge. Overall, among both Arabs and Jews, a high percentage, rarely or never try to learn about the coverage of SHI (72% vs. 67%) (Data is not shown in a table).

## Discussion

The current study examined differences in SHI knowledge and understanding between two population groups in Israel- Arabs and Jews. Findings show that overall, owning SHI was substantially higher among Jews. In general, about a third of both groups did not investigate what SHI covers before purchasing it and relatively few knew exactly what it cost. There were misconceptions about what SHI covers and about the need for SHI for general health care. After controlling for selected confounding variables, such as socio-demographic variables (gender, age and education) and SHI ownership, differences between Arabs and Jews in SHI knowledge and understanding persist.

SHI knowledge was lower among Arabs compared to Jews, when measured by objective measures, as well as by participants’ subjective self-reports about their lack of knowledge. The lower SHI knowledge found among Arabs coincides with lower rates of SHI ownership and lower SES, as well as with previous research, suggesting lower levels of knowledge and awareness to health related issues is a result of socioeconomic gaps [14].

The lower scores in SHI knowledge among Arabs may be a marker of less efficient use of healthcare services. In a study conducted in the United States, the authors pointed out that each person must understand and select the most appropriate plan, while understanding which services are and are not covered [14]. In a systematic review, beneficiaries’ knowledge of the United States optional Part D program for Medicaid was poor, particularly with regards to the coverage gap and the low-income subsidy [15], and it has been found that consumers tend to make decisions on the basis of anecdotal information such as their friends’ experiences [16]. There is evidence that this type of information can result in a biased selection of SHI [17].

The gaps found in the current study between Arabs and Jews in knowledge and understanding of SHI may be due, in part, to the cultural differences between the two population groups. Arabs in Israel are just one of the groups that are in risk to have low level of knowledge and understanding of health insurance. Others could include the elderly and other ethnic minorities [18–20]. While low level knowledge and understanding in SHI may lead to difficulties in operating in the insurance market, these difficulties can cause frustration and disappointment (for example, from unanticipated out of pocket expenses) and to lack of use and exposure to health care services, which then reinforce the low health insurance knowledge and understanding.

In the current study, among the sample of Jews, respondents reported obtaining most of their information on medical issues, including SHI, on the internet. In the sample of Arabs, respondents reported obtaining most of the information from physicians or television and radio. Since most of the information today is received online and since doctors are limited in the amount of time they can spend with patients, those not using the internet may get a restricted view of the services and treatments suitable to their medical needs. On the other hand, information available online can be overwhelming and sometimes misleading. This might lead to wrong assumptions and expectations.

The success of the health insurance market depends greatly on consumers’ ability to understand SHI and to make informed decisions [21, 22]. Thus, health insurance knowledge and understanding depends on obtaining valid and accurate information. Our findings show that both Arabs and Jews expected full coverage for services that had only partial coverage, such as surgical operations abroad, choosing a specialist or a surgeon and consultation with a specialist. It seems that the low level of SHI knowledge had an effect on expectations by creating confusion regarding different services and the insurance co-payments. Apart from low level of knowledge, another possible explanation of these high expectations is the aggressive marketing by SHI and commercial insurance companies, while trying to recruit new costumers.

These findings may also reflect socio-cultural differences between the two ethnic groups and suggest the importance of providing services adapted to different population groups. It also suggests that lack of familiarity with SHI services may influence preferences and expectations from SHI.

Our findings also show that 35% of the Jews and 52.5% of the Arabs who had SHI, declared that they had either never used or had no recollection of using SHI. The gaps found in utility of SHI between Arabs and Jews may reflect the gaps in SHI knowledge and understanding. These findings are in accordance with previous literature about the relationship between lack of utility and lack of knowledge and understanding of health insurance [23].

The current research shows that in the Israeli SHI market, consumers’ behavior isn’t necessarily rational, partly because it isn’t based on a complete understanding of SHI. Regardless of the socio-economic gaps between Arabs and Jews, both population groups had high expectations and low knowledge and understanding regarding SHI services. Consumers were expecting to receive coverage for more services than SHI covers in practice.

The information from the current study can assist in identifying some of the barriers and contributing factors to knowledge and understanding of supplemental health insurance among populations at risk and provide information regarding groups that will benefit from support in their decision on purchasing SHI or operating it.

Tools for measuring health insurance literacy have recently been reported [19, 21] and this is an important area for future research.

### Potential limitations of the study

The samples for the population survey were selected at random. However, the use of random digit dialing restricted the results of the surveys to those with land lines resulting in selection bias. This does not seem to be differentially associated with the measures of SHI knowledge and understanding between Arabs and Jews. However, this factor should be borne in mind when considering the generalizability of the findings. Since the data is based on self-reports, there may be information bias. However, there is no reason to believe that the size of the bias would be differentially distributed between the variables in any of the dependent-independent variable association examined. Thus, there may be an attenuation of the associations detected. While the most likely confounding variables were controlled in multiple regression analysis, there may be residual confounding or information bias in the confounding variables that remained uncontrolled.

## Conclusions

This study demonstrates poor knowledge and understanding of the services offered by SHI in the whole Israeli population. We provide information regarding groups who should benefit from additional support and guidance while making decisions about purchasing SHI. One way to provide information and education is through general practitioners and nurses employed by the health providers. The gaps between Arabs and Jews in SHI knowledge and understanding imply that the type of information and the way it is disseminated should be adapted to different population groups.

## Additional file

Additional file 1: Health Insurance Questionnaire. (DOC 109 kb)

## Abbreviations

CHI: Commercial Health Insurance
CI: Confidence intervals
HMO: Health Maintenance Organization
MOH: Ministry of Health
NGO: Non Governmental Organization
NHI: National Health Insurance
NIS: New Israeli Shekels
SES: Social Economic Status
SHI: Supplemental Health Insurance
