# Supplementary material for: A national survey of ethnic differences in knowledge and understanding of supplementary health insurance
Source: Isr J Health Policy Res. 2017 Mar 7;6:12. doi: 10.1186/s13584-017-0137-4 (PMC5340019; doi:10.1186/s13584-017-0137-4)
Supplement: Additional file 1: — Health Insurance Questionnaire. (DOC 109 kb) [file 13584_2017_137_MOESM1_ESM.doc]

**Appendix 1: Health Insurance Questionnaire**

1. Year of birth

[If the person is out of the age limits, please ask: May I speak to someone between ages 25-75?]

1. Do you have supplementary health insurance (SHI) throw your health care provider?
2. Yes (Skip the next question)
3. No, but I had it before
4. I never had SHI
5. Don’t know/ Don’t remember
6. If not, why don’t you have SHI? [Read options out loud]
7. The basic health insurance provides all the necessary services
8. I have private health insurance
9. I find SHI too expensive
10. Other

The following questions refer to participants who've answered “yes” in question 2. If answered no- skip to question number 14]

1. Who is paying for the health insurance in your home?
2. Me
3. My parents
4. My children
5. Other
6. Do you know how much is your monthly payment for this insurance?
7. Know precisely
8. Approximately
9. Don’t know
10. How much do you pay each month for this insurance: |__|__||__|
11. To what extent is SHI payment come on the expense of other expenses?
12. Not at all
13. A little
14. sometimes
15. much
16. Very much
17. Don’t know
18. Have you ever used the SHI benefits?
19. Yes, in the recent year
20. Yes, but not in the recent year
21. I never used SHI benefits
22. Don’t know
23. Before purchasing have you checked what’s covered by the SHI?
24. Yes
25. No
26. Not relevant (purchase made by someone ells)

Please rate how much you agree with the following statements from 1-5, 5 being “agree” and 1 being “disagree”:

|  |  | **disagree** |  |  |  | **agree** |
| --- | --- | --- | --- | --- | --- | --- |
|  | I have SHI because of shorter waiting lists for doctors and treatments. | 1 | 2 | 3 | 4 | 5 |
|  | I have SHI for better high standard treatment | 1 | 2 | 3 | 4 | 5 |
|  | I have SHI because of fear from catastrophic illnesses not covered by the basic health insurance | 1 | 2 | 3 | 4 | 5 |
|  | I have SHI for subsidized medications and treatments | 1 | 2 | 3 | 4 | 5 |

1. Have you ever changed your HMO provider?
2. Yes
3. No (skip the next question)
4. Have you ever changed your HMO provider for better SHI?
5. Yes
6. No

Please note which of the following medical services are covered by your SHI:

| 1. Subsidized Private nurse in hospitalization after surgery | 1. Included | 2. Not Included | 3. Don’t know |
| --- | --- | --- | --- |
| 1. Choosing a doctor or a surgeon from every hospital in Israel | 1. Included | 2. Not Included | 3. Don’t know |
| 1. Subsidized private prenatal care and fertility treatments | 1. Included | 2. Not Included | 3. Don’t know |
| 1. Subsidized transplants and surgeries abroad | 1. Included | 2. Not Included | 3. Don’t know |
| 1. Private room in different hospitals in Israel | 1. Included | 2. Not Included | 3. Don’t know |
| 1. Cancer Medications not covered by basic health insurance | 1. Included | 2. Not Included | 3. Don’t know |
| 1. Partial coverage of Consultation with a specialist | 1. Included | 2. Not Included | 3. Don’t know |
| 1. Full coverage of Private physiotherapy | 1. Included | 2. Not Included | 3. Don’t know |

In order to keep a reasonable cost for the SHI, not all medical services can be included. Please rate how important is it for you that the SHI will cover each service, between 1-5, 5 being very important and 1 being not at all important:

|  | **Not at all important** |  |  |  | **Very important** |
| --- | --- | --- | --- | --- | --- |
| 1. Choosing a doctor or a surgeon | 1 | 2 | 3 | 4 | 5 |
| 1. Consultation with a specialist | 1 | 2 | 3 | 4 | 5 |
| 1. Private room in hospital | 1 | 2 | 3 | 4 | 5 |
| 1. Cancer Medications not in the basic package | 1 | 2 | 3 | 4 | 5 |
| 1. Full coverage of transplants and surgeries abroad | 1 | 2 | 3 | 4 | 5 |

1. [If have SHI] what is the most important reason for you purchasing SHI? [choose one option only]
2. better high standard treatment
3. subsidized medications and treatments
4. Shorter waiting lists for doctors and treatments.
5. fear from catastrophic illnesses not covered by the basic health insurance
6. other
7. don’t know (purchase made by someone ells)
8. [If have SHI] what is the least important reason for you purchasing SHI? [choose one option only]
9. better high standard treatment
10. subsidized medications and treatments
11. Shorter waiting lists for doctors and treatments.
12. fear from catastrophic illnesses not covered by the basic health insurance
13. other
14. don’t know (purchase made by someone ells)
15. Do you have a private health insurance through your work place?
16. Yes
17. No [skip the next question]
18. Don’t know/don’t remember [skip the next question]
19. [if answered yes] if you didn’t have work health insurance, would you purchase private health insurance yourself?
20. Yes
21. No
22. Don’t know
23. Do you have private health insurance throw an insurance company like: Menora, AIG, Harel ext..?
24. Yes
25. No [skip the next 5 questions]
26. Don’t know/remember [skip the next 5 questions]

| 1. Have you purchase PI for Medication not covered by basic package? | 1. yes | 2. no | 3. Don’t know |
| --- | --- | --- | --- |
| 1. Have you purchase PI because of fear from catastrophic illnesses not covered by the basic health insurance? | 1. yes | 2. no | 3. Don’t know |
| 1. Have you purchase PI for choosing the physician and the hospital? | 1. yes | 2. no | 3. Don’t know |
| 1. Have you purchase PI for better high standard treatment? | 1. yes | 2. no | 3. Don’t know |
| 1. Have you purchase PI because you don’t trust the SHI? | 1. yes | 2. no | 3. Don’t know |

How do you usually get information about events related to health, health insurance, disease prevention and treatment?

|  | **Not at all** |  |  |  | **Very often** |
| --- | --- | --- | --- | --- | --- |
| 1. Newspaper | 1 | 2 | 3 | 4 | 5 |
| 1. Internet/Email | 1 | 2 | 3 | 4 | 5 |
| 1. Radio and television | 1 | 2 | 3 | 4 | 5 |
| 1. Family members, friends, co-worker | 1 | 2 | 3 | 4 | 5 |
| 1. Physician / nurse | 1 | 2 | 3 | 4 | 5 |

1. How often do you read information regarding SHI?
2. Not at all
3. Rarely
4. Sometimes
5. Often
6. Very often
7. How much do you understand the doctor’s/nurse’s instructions?

| Very much | 5 | 4 | 3 | 2 | 1 | Not at all |
| --- | --- | --- | --- | --- | --- | --- |

1. Do you have any information missing about your SHI?
2. Yes
3. No

**General questions:**

1. Compared to your age group, how would you rate your overall health?

| excellent | 5 | 4 | 3 | 2 | 1 | poor |
| --- | --- | --- | --- | --- | --- | --- |

1. Do you have any chronic illnesses?
2. Yes
3. No
4. Do you take medications on a regular basis?
5. Yes
6. No

49a) Are you currently smoking cigarettes?

1. Yes
2. No
3. Gender:
4. Male
5. Female
6. Country of birth:
7. Israel [skip the next question]
8. Other
9. If other, then year of immigration:|__|__|__||__
10. What is your native tongue?
11. Hebrew (go to question 62)
12. Arabic (go to question 56)
13. Russian (go to question 60)
14. Other (go to question 54)

| If “other” | Very poor |  |  |  | excellent |
| --- | --- | --- | --- | --- | --- |
| 1. Please indicate your ability to read Hebrew | 1 | 2 | 3 | 4 | 5 |
| 1. Please indicate your ability to understand Hebrew | 1 | 2 | 3 | 4 | 5 |

| If “Arabic” | Very poor |  |  |  | excellent |
| --- | --- | --- | --- | --- | --- |
| 1. Please indicate your ability to read Hebrew | 1 | 2 | 3 | 4 | 5 |
| 1. Please indicate your ability to understand Hebrew | 1 | 2 | 3 | 4 | 5 |

| If “Russian” | Very poor |  |  |  | excellent |
| --- | --- | --- | --- | --- | --- |
| 1. Please indicate your ability to read Hebrew | 1 | 2 | 3 | 4 | 5 |
| 1. Please indicate your ability to understand Hebrew | 1 | 2 | 3 | 4 | 5 |
| 1. Please indicate your ability to read Russian | 1 | 2 | 3 | 4 | 5 |
| 1. Please indicate your ability to understand Russian | 1 | 2 | 3 | 4 | 5 |

1. What has your occupational status been during the past year:
2. Salaried employment
3. Self-employed
4. Pensioner
5. Housewife
6. Student
7. Not working/unemployed
8. Other
9. Marital Status:
10. Single
11. Currently married or with a life partner
12. Divorced/separated/single parent
13. Widowed
14. Number of children under 18: |__|__|
15. Number of persons generally reside at your home (including you): |__|__|
16. [If resides alone] the average net income per person is currently 8000 NIS. Is your total monthly net income:
17. Below the average net income
18. Like the average net income
19. Above the average net income
20. Don’t know
21. [if do not resides alone]] The average net income per household is 12,000.is the total net income of your household:
22. Below the average net income
23. Like the average net income
24. Above the average net income
25. Don’t know
26. What is your educational level:
27. Primary school.
28. High school education without matriculation certificate
29. High school education with matriculation certificate
30. Academic degree
31. Religious studies
32. Other
